# Effect of saroglitazar 2 mg and 4 mg on glycemic control, lipid profile and cardiovascular disease risk in patients with type 2 diabetes mellitus: a 56-week, randomized, double blind, phase 3 study (PRESS XII study)

**DOI:** 10.1186/s12933-020-01073-w

**Published:** 2020-06-19

**Authors:** Manjunath Krishnappa, Kishor Patil, Krupi Parmar, Purav Trivedi, Nirali Mody, Chintan Shah, Khushboo Faldu, Sanjay Maroo, for the PRESS XII study group, Piyush Desai, Kamlesh Fatania, Satyanarayan Murthy, R. Balamurugan, Manish Agarwal, K. P. Singh, G. S. Kalra, Vipul Khandelwal, Ashish Singwala, Hemant Thacker, Rahul Tulle, Harish Rao, Mukund Kumbla, Parminder Singh, Ashok Khatri, Sumit Agrawal, R. N. Sarkar, Dinesh Agarwal, Girish Bhatia, R. P. Agarwal, Surender Kumar, P. R. Vamsi Krishna, Ajay Kumar Ajmani, Amit Asalkar, Indraneel Basu, Sudip Chatterjee, Vinod Kumar Pavithran, Rupam Das, Aniruddha Dharmadhikari, Vikram Vardhan, M. Madusudhan Babu, Nilanjan Sengupta, Srirang Abkari, R. Harikrishna, Rashmi Chovatia, Deven Parmar

**Affiliations:** 1 Zydus Research Centre, Clinical R & D, Cadila Healthcare Limited, Sarkhej-Bavla N. H. No. 8 A, Moraiya, Ahmedabad, Gujarat 382213 India; 2 Zydus Discovery DMCC, Dubai, UAE

**Keywords:** Type 2 diabetes mellitus, Saroglitazar, Peroxisome proliferator-activated receptors (PPAR α/γ) agonist, Pioglitazone, Cardiovascular disease risk

## Abstract

**Background:**

The potential for PPAR agonists to positively affect risk of cardiovascular disease in patients with type 2 diabetes (T2DM) is of persistent attention. The PRESS XII study primarily aimed to evaluate the efficacy and safety of saroglitazar (2 mg and 4 mg) as compared to pioglitazone 30 mg on glycemic control in patients with type 2 diabetes mellitus.

**Methods:**

In this randomized double-blind study, patients with T2DM [glycosylated hemoglobin (HbA1c) ≥ 7.5%] were enrolled from 39 sites in India. Patients received once-daily doses of either saroglitazar or pioglitazone (1:1:1 allocation ratio) for a total of 24 weeks. Patients were continued in a double blind extension period for an additional 32 weeks. Efficacy evaluations of glycemic parameters [HbA1c (Primary endpoint at week 24), FPG and PPG] and other lipid parameters (TG, LDL-C, VLDL-C, HDL-C, TC, Non HDL-C, Apo A1 and Apo B) were conducted at week 12, 24 and 56 and compared to the baseline levels. The efficacy analyses were performed by using paired t-test and ANCOVA model.

**Results:**

A total of 1155 patients were enrolled in this study. The baseline characteristics were similar between the three treatment groups. The within group mean (± SD) change in HbA1c (%) from baseline of the saroglitazar (2 mg and 4 mg) and pioglitazone treatment groups at week 24 were: − 1.38 ± 1.99 for saroglitazar 2 mg; − 1.47 ± 1.92 for saroglitazar 4 mg and − 1.41 ± 1.86 for pioglitazone, respectively. Statistically significant reduction from baseline in HbA1c was observed in each treatment group at week 24 with p-value < 0.016. There was a significant reduction in TG, LDL-C, VLDL-C, TC and Non HDL-C with a significant increase in HDL-C from baseline levels (< 0.016). Most of the AE’s were ‘mild’ to ‘moderate’ in severity and were resolved by the completion of the study.

**Conclusions:**

Saroglitazar effectively improved glycemic control and lipid parameters over 56 weeks in patients of T2DM receiving background metformin therapy and has a promising potential to reduce the cardiovascular risk in T2DM patients.

*Trial registration* CTRI/2015/09/006203, dated 22/09/2015

## Background

Type 2 diabetes mellitus (T2DM) is a complex metabolic disorder characterized by persistent hyperglycaemia due to relative insulin deficiency, insulin resistance, dyslipidemia and vascular inflammation that are associated with an increase in the risk for cardiovascular diseases (CVDs) [1, 2]. An estimated 463.0 million adults aged 20–79 years have diabetes across the world [3]. In 2019, 77 million patient had diabetes in India, which will increase to 101.0 million and 134.2 million by year 2030 and 2045, respectively [3]. Due to a high prevalence of T2DM in India coupled with a genetic predisposition to develop CVD as supported by few genome-wide association studies [4, 5], the incidence of CVDs have been increasing in the Indian population, and are the leading causes of morbidity and mortality in India [6–9]. Because of the CVD risk, drug therapies improving glycemic control as well as the lipid parameters have become progressively important in the management of T2DM patients [10]. As per the recommendations from clinical guidelines, the CVD risk in diabetes can be improved by treating dyslipidaemia and hyperglycaemia. However, a majority of the patients still do not attain the recommended goals for these risk factors [11, 12].

Peroxisome proliferator-activated receptors (PPAR α and PPAR γ) are important for regulating glucose and lipid metabolism [13]. Many PPAR-α/γ agonists were taken up for clinical development over the past two decades, however, due to lack of efficacy or safety issues many of them have been discontinued from the clinical development programs and have not progressed beyond the phase III development stage [14, 15]. Nonetheless, in spite of such failures, many newer PPAR agonists are still being taken up for clinical development considering the potential benefits they can offer keeping in mind their mechanistic actions on the lipid and glucose metabolism. PPAR agonists that are capable of activating both α and γ PPAR receptors can concurrently ameliorate glycaemic control as well as lipid abnormalities typically observed in T2DM patients [16].

Saroglitazar is a novel dual PPAR α/γ agonist having strong PPAR-α effect with moderate PPAR-γ effect. Saroglitazar is already approved in India since 2013 for diabetic dyslipidemia and hypertriglyceridemia with T2DM not controlled by statin therapy [17, 18]. Recently (January 2020), saroglitazar received approval for marketing as an add-on therapy to metformin for treatment of type 2 diabetes mellitus in India. It also received approval for marketing in India for Non cirrhotic Non-alcoholic steatohepatitis in March 2020. Saroglitazar is the first and only dual PPAR α/γ agonist among the glitazars to be approved as well as prescribed extensively in clinical practice, anywhere in the world. Compared with other PPAR-α/γ agonists (Such as muraglitazar, tesaglitazar and aleglitazar), during the entire clinical development program, there were no safety concerns with saroglitazar, especially weight gain and edema. Additionally, as evident from the preclinical and clinical data, saroglitazar exhibited beneficial effects not only on the lipid profile but also on glycemic control for the treatment of dyslipidemia and diabetes [19–21].

Pioglitazone is widely used in clinical practice to lower blood glucose levels in subjects with T2DM. It acts as an agonist for PPAR-γ activated nuclear transcription factors that modulate gene expression, lowering blood glucose primarily by increasing insulin sensitivity in peripheral tissues. It offers control of hyperglycaemia as well as provides favourable improvements in high-density lipoprotein cholesterol (HDL-C) and triglyceride (TG) levels [22–25]. This randomized study was designed to evaluate the efficacy and safety of saroglitazar 2 mg and 4 mg as compared to pioglitazone 30 mg on glycemic control in patients with type 2 diabetes mellitus over a 56-week study duration, who were receiving background metformin therapy.

## Methods

### Study design

**Fig. 1:**
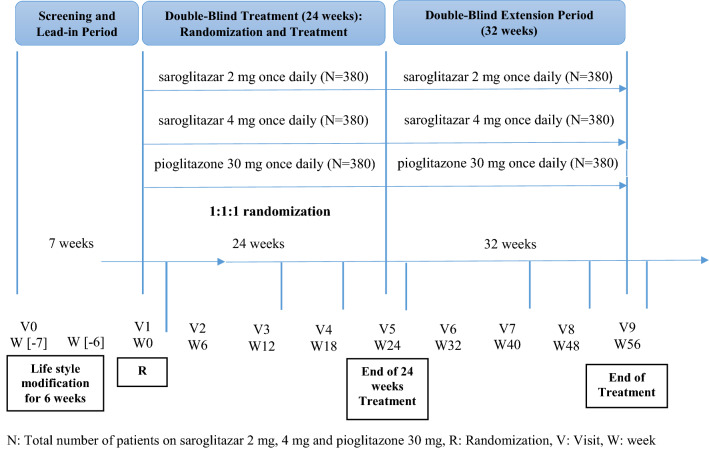
Study design

This was a multi-centric, prospective, randomized, double-blind, active-control, phase 3 study conducted at 39 sites in India and was registered with the Clinical Trial Registry of India (CTRI/2015/09/006203). The study consisted of a 6 week lead-in/run-in period, followed by a double-blind active-controlled treatment period for 24 weeks and a 32 week double blind extension period (Fig. 1). Eligible patients were randomly assigned in a 1:1:1 treatment allocation ratio to saroglitazar 2 mg, saroglitazar 4 mg, and pioglitazone. The study was conducted in accordance with the ethical principles of the Declaration of Helsinki and guidelines laid down by the International Council for Harmonisation (ICH)-Good Clinical Practice (GCP), Central Drugs Standard Control Organization (CDSCO), ethical guidelines for biomedical research on human subjects issued by Indian Council of Medical Research (ICMR) and regulations/guidelines of the Government of India. The institutional review board of each center approved the protocol, and all the patients provided written informed consent before participation in the study.

### Participants

**Fig. 2:**
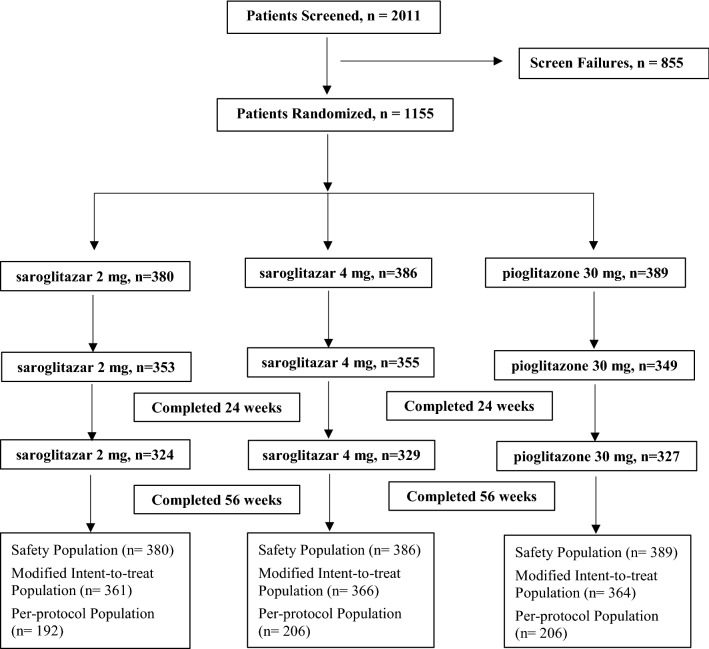
Patient disposition

Patients were recruited from 8 October 2015 to 25 June 2018 from different hospital clinics across India. We enrolled 1155 (saroglitazar 2 mg: 380, saroglitazar 4 mg: 386, and pioglitazone 30 mg: 389) T2DM patients aged 18 to 75 years of either sex as per the American Diabetes Association (ADA) criteria (Fig. 2). After a lifestyle modification of 6 weeks, patients had to meet the following inclusion criteria: history of type 2 diabetes mellitus [glycosylated hemoglobin (HbA 1c) ≥ 7.5%.], history of stable metformin dose at least since last 6 weeks (total daily dose not exceeding 2 gm) in conjunction with diet and exercise, and fasting plasma glucose (FPG) ≤ 270 mg/dL (14.98 mmol/L).

The patients were excluded if they had been treated with insulin for ≥ 7 days within the past 3 months prior to the screening visit and glitazone/glitazar therapy in the past 1 month, had type 1 diabetes mellitus or a history of ketoacidosis or secondary forms of diabetes, had a history of recurrent or severe hypoglycemia within the last 3 months, history of acute or chronic metabolic acidosis (including diabetic ketoacidosis), history of unstable or rapidly progressive diabetic retinopathy, nephropathy (serum creatinine > 1.5 mg/dL), had coronary insufficiency (e.g., a myocardial infarction, a coronary angioplasty or bypass graft, unstable angina, transient ischemic attacks, or a documented cerebrovascular accident within 6 months prior to the screening visit). Patients with cardiac failure or history of cardiac failure [New York Heart Association (NYHA) Stages 3 to 4], uncontrolled hypertension [blood pressure (BP) > 160/100 mmHg] and alanine transaminase (ALT) level ≥ 2.5 times the upper normal limit (UNL), active liver disease, or jaundice were also excluded. Women who were pregnant or breastfeeding were also excluded from the study.

### Procedures

At the beginning of the study, patients underwent a life style modification for 6 weeks before participating in the study. Following screening of the patients, the eligible patients entered into a 6-week lead-in/run-in period. During this period, subjects stopped any other antidiabetic agents and remained on their current dose of metformin.

Following the 6-week run-in period, patients visited the site at baseline where randomization was performed (week 0). They had to take either saroglitazar 2 mg, or saroglitazar 4 mg, or pioglitazone once daily in the morning before breakfast for a period of 56 weeks (24 weeks double-blind active control period and 32 weeks optional extension period) (Fig. 1).

At week 24, patients who completed the double-blind period, had the option of entering the double-blind extension study in which they received saroglitazar 2 mg, or saroglitazar 4 mg or pioglitazone with their standard regimen of metformin.

This was a double blind randomized study, where both the Investigator as well as the subjects were not aware of the treatment being administered. Furthermore, the randomization scheme was not available to the clinic staff, study monitors and/or other individuals involved in the study. The randomization scheme was generated by using SAS^®^ statistical software (Version: 9.4; SAS Institute Inc., USA). A scratch card system was used for blinding of the randomization allocation. The study medication compliance was verified by the study sites by examination of the container and tablet counts.

### Assessments

The primary efficacy endpoint was change from baseline in HbA1c for saroglitazar 2 mg, saroglitazar 4 mg and pioglitazone at week 24. The secondary efficacy endpoints were:

- the change from baseline in HbA1c at week 12 and week 56, the change from baseline in FPG, 2 h postprandial plasma glucose (PPG), triglycerides (TG), low density lipoprotein cholesterol (LDL-C), very low density lipoprotein cholesterol (VLDL-C), high density lipoprotein cholesterol (HDL-C), total cholesterol (TC), Non-HDL Cholesterol (Non HDL-C), apolipoprotein A1 (Apo A1), and apolipoprotein B (Apo B) at week 12, week 24 and week 56;
- Comparison of change from baseline in HbA1c and FPG between saroglitazar 2 mg, saroglitazar 4 mg and pioglitazone at week 12, week 24 and week 56.

Safety was evaluated by physical examination, adverse event monitoring, cardiac function (2D ECHO and ECG), vital sign measurements, and laboratory assessments. All the serious adverse events were reported to the IRB’s as well as the Drug Controller General of India. A DSMB comprising independent subject matter experts and a statistician held meetings to review the safety aspects of the study. All the laboratory analysis was performed using standard methods at APL Institute of Clinical Laboratory & Research Pvt. Ltd (Ahmedabad, India), which is accredited by the National Accreditation Board for Testing and Calibration Laboratory.

### Statistical analysis

#### Sample size justification

##### Sample size justification for change from baseline in each treatment group

Sample size of 85 subjects achieved 90% power to detect a mean of paired differences of 0.3 HbA1c with a known standard deviation of differences of 0.9 and with 5% significance level.

##### Sample size justification for comparison of saroglitazar vs. pioglitazone

Sample size of 342 subjects in each group was required to achieve 80% power with a 2.5% level of significance considering non-inferiority margin (− 0.20), difference in change from baseline in HbA1c at week 24 for saroglitazar 2 mg and 4 mg vs. pioglitazone (~ − 0.5%) and standard deviation (0.7).

For the primary efficacy endpoint of change from baseline, the baseline values were compared to post baseline values using paired t-test.

For the secondary efficacy endpoints, treatment effect was evaluated using an analysis of co-variance (ANCOVA) model with baseline as co-variate and treatments factor. Treatment effect was estimated using the least-square means and 95% confidence intervals (CIs) from the ANCOVA model. Pair-wise comparison was done for saroglitazar 2 mg or 4 mg versus pioglitazone. To establish non-inferiority of test products (saroglitazar 2 mg or 4 mg) over pioglitazone for mean change from baseline in HbA1c, the lower bound of the 2-sided 95% CI is to be greater than or equal to non-inferiority margin. In this study, the per-protocol population was considered definitive for the primary efficacy analysis while the modified intent-to-treat population was considered supportive.

All secondary laboratory parameters were analyzed in the same manner as the primary parameter. Safety parameters were analyzed descriptively. The SAS^®^ package (SAS^®^ Institute Inc., USA, and Version 9.4) was used for statistical evaluation. Data are presented as mean ± standard deviation (SD). For all statistical analyses, *p* < 0.016 was considered statistically significant.

## Results

### Study population

A total of 1155 patients were enrolled and assigned randomly in a ratio of 1:1:1 to the treatment groups (saroglitazar 2 mg, saroglitazar 4 mg and pioglitazone). Of these, 980 patients completed the study up to Visit 9 (week 56). Disposition of the patients in this study is presented in Fig. 2. The baseline characteristics were similar between the three treatment groups. The characteristics of the patient population at baseline are presented in Table 1.

**Table 1:** Baseline characteristics

	Saroglitazar 2 mg (N = 380)	Saroglitazar 4 mg (N = 386)	Pioglitazone 30 mg (N = 389)
Subjects characteristics			
Age (years), m ± SD	51.90 ± 10.38	51.34 ± 10.06	51.84 ± 9.76
Female, n (%)	164 (43.16%)	143 (37.05%)	167 (42.93%)
Male, n (%)	216 (56.84%)	243 (62.95%)	222 (57.07%)
Weight (kg), m ± SD	70.27 ± 11.84	69.09 ± 11.46	69.49 ± 11.59
BMI (kg/m^2^), m ± SD	26.48 ± 4.03	25.94 ± 3.87	26.33 ± 4.07
Glycemic parameters^a^, m ± SD			
HbA_1C_ (%)	9.76 ± 1.59	9.72 ± 1.58	9.49 ± 1.54
FPG (mg/dL)	166.08 ± 46.14	165.41 ± 51.39	165.08 ± 51.45
PPG (mg/dL)	275.90 ± 84.74	277.42 ± 90.57	277.35 ± 88.05
Lipid parameters^a^, m ± SD			
TG (mg/dL)	163.87 ± 91.49	172.52 ± 123.67	166.20 ± 89.93
LDL-C (mg/dL)	117.11 ± 36.92	112.93 ± 34.89	116.77 ± 32.31
VLDL-C (mg/dL)	32.77 ± 18.30	34.50 ± 24.73	33.24 ± 17.99
HDL-C (mg/dL)	42.39 ± 10.58	41.50 ± 10.47	42.64 ± 12.72
TC (mg/dL)	176.98 ± 42.67	174.03 ± 39.32	176.42 ± 37.83
Non HDL-C (mg/dL)	134.62 ± 41.06	132.54 ± 39.12	133.78 ± 35.39
Apolipoproteins^a^, m ± SD			
Apo A1 (mg/dL)	128.72 ± 23.87	124.89 ± 23.05	127.15 ± 22.80
Apo B (mg/dL)	100.64 ± 29.77	98.79 ± 26.06	100.60 ± 23.07

*Apo* apolipoprotein A1, *Apo B* apolipoprotein B, *BMI* body mass index, *dL* decilitre, *FPG* fasting plasma glucose, *HbA1c* glycosylated hemoglobin, *HDL-C* high-density lipoprotein cholesterol, *LDL-C* low-density lipoprotein cholesterol, *mg* milligram, *m* mean, *n* number, *N* number of patients in each treatment group, *PPG* postprandial plasma glucose, *SD* standard deviation, *TC* total cholesterol, *TG* triglyceride, *VLDL-C* very low-density lipoprotein cholesterol

^a^Represents baseline value for per-protocol population

### Glycemic control

**Fig. 3:**
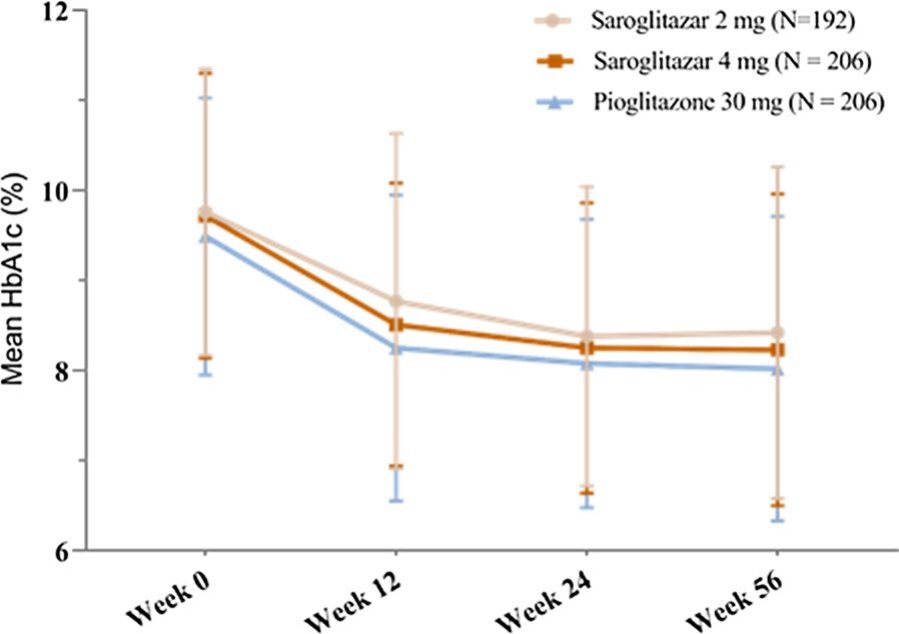
Reduction in mean HbA1c levels during 56 weeks treatment

The primary endpoint of the study was change from baseline in HbA1c for saroglitazar 2 mg, 4 mg and pioglitazone at week 24 (Within treatment group comparison). The within group mean (± SD) change in HbA1c (%) from baseline of the saroglitazar (2 mg and 4 mg) and pioglitazone treatment groups at week 24 were: − 1.38 ± 1.99 for saroglitazar 2 mg; − 1.47 ± 1.92 for saroglitazar 4 mg and − 1.41 ± 1.86 for pioglitazone, respectively. There was a consistent reduction in mean HbA1c levels from week 12 to week 56 (Fig. 3). Statistically significant reduction from baseline in HbA1c was observed in each treatment group at week 24 with p-value < 0.016 (Table 2).

**Table 2:** Absolute change in glycemic parameters at week 12, week 24, and week 56 from baseline

Efficacy outcome	Laboratory assessment at study time point	Saroglitazar 2 mg (n = 192) m ± SD	Saroglitazar 4 mg (n = 206) m ± SD	Pioglitazone 30 mg (n = 206) m ± SD
HbA_1C_ (%)	Absolute change at week 12	− 0.99 ± 1.84*	− 1.21 ± 1.85*	− 1.25 ± 1.88*
	Absolute change at week 24	− 1.38 ± 1.99*	− 1.47 ± 1.92*	− 1.41 ± 1.86*
	Absolute change at week 56	− 1.34 ± 2.01*	− 1.49 ± 1.97*	− 1.47 ± 2.01*
FPG (mg/dL)	Absolute change at week 12	− 2.93 ± 73.34	− 9.23 ± 70.73	− 15.46 ± 69.68*
	Absolute change at week 24	− 0.09 ± 72.72	− 8.09 ± 78.76	− 12.70 ± 67.98*
	Absolute change at week 56	− 17.13 ± 62.04*	− 17.19 ± 70.29*	− 21.13 ± 65.02*
PPG (mg/dL)	Absolute change at week 12	− 33.59 ± 107.06*	− 42.86 ± 102.22*	− 48.60 ± 100.38*
	Absolute change at week 24	− 35.46 ± 108.81*	− 44.36 ± 103.73*	− 45.52 ± 101.73*
	Absolute change at week 56	− 45.98 ± 106.45*	− 43.33 ± 97.54*	− 51.33 ± 107.36*

Absolute change at week 12 = value at week 12 − value at baseline

Absolute change at week 24 = value at week 24 − value at baseline

Absolute change at week 56 = value at week 56 − value at baseline

Primary efficacy endpoint was the absolute change from baseline to week-24 in HbA1c for saroglitazar 2 mg, saroglitazar 4 mg and pioglitazone 30 mg

*dL* decilitre, *FPG* fasting plasma glucose, *HbA1c* glycosylated hemoglobin, *mg* milligram, *m* mean, *n* number of patients, *PPG* postprandial plasma glucose, *SD* standard deviation

* Significant difference compared to baseline using paired t-test (*p* value < 0.05 which is < 0.016 for each treatment group)

Statistically significant reduction from baseline in fasting plasma glucose was observed at week 12, week 24 and week 56 in pioglitazone treatment group and at week 56 in saroglitazar 2 mg and 4 mg treatment groups with p-value < 0.016.

The saroglitazar (2 mg and 4 mg) and the pioglitazone (30 mg) treatment groups showed statistically significant reduction in 2 h PPG at week 12, 24 and 56 with p-value < 0.016.

### Comparison with pioglitazone (between treatment group comparisons)

One of the secondary endpoints in the study was comparison of change from baseline HbA1c between saroglitazar 2 mg and 4 mg with pioglitazone. In the comparison between saroglitazar 4 mg and pioglitazone, the lower bound of two-sided 95% CI was found to be greater than the predefined non-inferiority margin of − 0.20 at week 12, 24 and 56. The 95% CI contains ‘0’ and p-value is statistically not significant at one-sided p-value of 0.025. Thus, leading to inference of ‘non-inferiority’ of saroglitazar 4 mg to pioglitazone at week 12, 24 and 56 (Table 3).

**Table 3:** Change from baseline in glycosylated hemoglobin and fasting plasma glucose between treatment groups

Efficacy outcome	Week	Treatment	n	Mean ± SD	LS mean difference of change from baseline saroglitazar vs. pioglitazone			
					Estimate	Standard error	95% confidence interval	p-values
HbA_1C_ (%)	week 12	Saroglitazar 2 mg	192	8.77 ± 1.86	0.40	0.17	(0.08, 0.73)	0.0156
		Saroglitazar 4 mg	206	8.51 ± 1.57	0.18	0.15	(− 0.12, 0.48)	0.2368
	week 24	Saroglitazar 2 mg	192	8.38 ± 1.66	0.23	0.16	(− 0.08, 0.54)	0.1536
		Saroglitazar 4 mg	206	8.25 ± 1.61	0.10	0.15	(− 0.19, 0.40)	0.4924
	week 56	Saroglitazar 2 mg	192	8.42 ± 1.84	0.31	0.17	(− 0.03, 0.65)	0.0698
		Saroglitazar 4 mg	206	8.23 ± 1.73	0.15	0.16	(− 0.18, 0.47)	0.3742
FPG (mg/dL)	week 12	Saroglitazar 2 mg	192	163.15 ± 70.14	13.31	6.26	(1.00, 25.61)	0.0341
		Saroglitazar 4 mg	206	156.18 ± 60.92	6.55	5.72	(− 4.69, 17.79)	0.2524
	week 24	Saroglitazar 2 mg	192	165.98 ± 67.44	13.28	6.24	(1.01, 25.54)	0.0340
		Saroglitazar 4 mg	206	157.32 ± 67.98	4.85	6.23	(− 7.39, 17.09)	0.4363
	week 56	Saroglitazar 2 mg	192	148.95 ± 52.41	4.76	5.18	(− 5.42, 14.93)	0.3587
		Saroglitazar 4 mg	206	148.22 ± 60.25	4.18	5.47	(− 6.57, 14.94)	0.4447

Estimate, standard error, p-values and 95% CI are calculated based on ANCOVA (Analysis of Covariance)

*n* number of subjects in the specified category, *LS* least square

In the comparison between saroglitazar 2 mg and pioglitazone, the lower bound of two-sided 95% CI was found to be greater than the predefined non-inferiority margin of − 0.20 at week 24 and 56. The 95% CI contains ‘0’ and p-value is statistically not significant at one-sided p-value of 0.025. Thus, leading to inference of ‘non-inferiority’ of saroglitazar 2 mg to pioglitazone at week 24 and 56 (Table 3).

In addition, one of the secondary endpoints in the study was comparison of change from baseline FPG between saroglitazar 2 mg and 4 mg with pioglitazone. In the comparison between saroglitazar 4 mg and pioglitazone, 95% CI contained ‘0’ with p-value > 0.025 implying that the observed difference is not statistically significant for saroglitazar 4 mg vs pioglitazone comparison at week 12, 24 and 56 (Table 3).

In the comparison between saroglitazar 2 mg and pioglitazone, 95% CI contained ‘0’ with p-value > 0.025 implying that the observed difference is not statistically significant for saroglitazar 2 mg vs pioglitazone comparison at week 56 (Table 3).

### Lipid parameters

#### Triglycerides

Statistically significant reduction from baseline in TG was observed at week 12, 24 and 56 in saroglitazar 4 mg treatment group, at week 12 in saroglitazar 2 mg treatment group and at week 24 in pioglitazone treatment group with a p-value < 0.016.

#### Low density lipoprotein cholesterol

Statistically significant reduction from baseline in LDL-C was observed at week 12, 24 and 56 in saroglitazar 2 mg treatment group and at week 24 and 56 in saroglitazar 4 mg treatment group and at week 56 in pioglitazone treatment group with a p-value < 0.016.

#### Very low density lipoprotein cholesterol

Statistically significant reduction from baseline in VLDL-C was observed at week 12, 24 and 56 in saroglitazar 4 mg treatment group, at week 12 in saroglitazar 2 mg treatment group and at week 24 in pioglitazone treatment group with a p-value < 0.016.

#### High density lipoprotein cholesterol

Statistically significant increase from baseline in HDL-C was observed at week 12 in saroglitazar 2 mg and saroglitazar 4 mg treatment groups with a p-value < 0.016.

#### Total cholesterol

Statistically significant reduction from baseline in total cholesterol was observed at week 12 in saroglitazar 2 mg treatment group and at week 24 in saroglitazar 4 mg treatment group with a p-value < 0.016.

#### Non-HDL cholesterol

Statistically significant reduction from baseline in Non-HDL-C cholesterol was observed at week 12 and 24 in saroglitazar 2 mg and 4 mg treatment groups with a p-value < 0.016.

### Apolipoproteins

#### Apolipoprotein A1

Statistically significant reduction from baseline in Apo A1 was not observed in any of the treatment groups.

#### Apolipoprotein B

Statistically significant reduction from baseline in Apo B was observed at week 12 in saroglitazar 2 mg and at Week 12 and 24 in saroglitazar 4 mg treatment group with p-value < 0.016.

The absolute mean changes from baseline in lipid and apolipoprotein parameters are presented in Table 4.

**Table 4:** Absolute change in lipid and apolipoprotein parameters at week 12, week 24, and week 56 from baseline

Efficacy outcome	Laboratory assessment at study time point	Saroglitazar 2 mg (n = 192) m ± SD	Saroglitazar 4 mg (n = 206) m ± SD	Pioglitazone 30 mg (n = 206) m ± SD
TG (mg/dL)	Absolute change at week 12	− 24.46 ± 91.48*	− 37.85 ± 119.74*	− 12.43 ± 104.28
	Absolute change at week 24	− 17.20 ± 125.30	− 40.09 ± 144.91*	− 18.81 ± 99.43*
	Absolute change at week 56	− 15.48 ± 117.09	− 28.19 ± 140.34*	− 17.16 ± 112.18*
LDL-C (mg/dL)	Absolute change at week 12	− 8.62 ± 33.82*	− 3.14 ± 37.62	− 2.97 ± 33.59
	Absolute change at week 24	− 10.11 ± 42.06*	− 12.49 ± 38.99*	− 5.60 ± 37.84*
	Absolute change at week 56	− 9.99 ± 45.25*	− 8.07 ± 42.08*	− 8.90 ± 40.80*
VLDL-C (mg/dL)	Absolute change at week 12	− 4.89 ± 18.30*	− 7.57 ± 23.95*	− 2.49 ± 20.86
	Absolute change at week 24	− 3.44 ± 25.06	− 8.02 ± 28.98*	− 3.76 ± 19.89*
	Absolute change at week 56	− 3.10 ± 23.42	− 5.64 ± 28.07*	− 3.43 ± 22.44*
HDL-C (mg/dL)	Absolute change at week 12	2.29 ± 11.61*	2.69 ± 10.35*	1.81 ± 12.85*
	Absolute change at week 24	2.23 ± 12.83*	0.92 ± 10.69	2.11 ± 13.65*
	Absolute change at week 56	2.15 ± 12.44*	1.98 ± 13.10*	2.09 ± 14.20*
TC (mg/dL)	Absolute change at week-12	− 8.30 ± 39.46*	− 6.00 ± 40.71*	− 0.47 ± 37.55
	Absolute change at week 24	− 6.31 ± 48.48	− 12.67 ± 42.22*	− 1.28 ± 44.83
	Absolute change at week 56	− 5.87 ± 54.19	− 6.43 ± 51.11	− 3.88 ± 46.01
Non HDL-C (mg/dL)	Absolute change at week 12	− 10.65 ± 39.28*	− 8.70 ± 41.20*	− 2.28 ± 38.09
	Absolute change at week 24	− 8.57 ± 46.30*	− 13.61 ± 41.83*	− 3.42 ± 44.69
	Absolute change at week 56	− 8.07 ± 53.57*	− 8.39 ± 51.01*	− 5.94 ± 45.66
Apo A1 (mg/dL)	Absolute change at week 12	0.24 ± 26.08	1.81 ± 24.18	− 1.40 ± 26.15
	Absolute change at week 24	− 1.81 ± 27.59	− 3.46 ± 25.00	− 3.17 ± 27.41
	Absolute change at week 56	1.37 ± 25.83	− 0.52 ± 27.09	0.63 ± 27.19
Apo B (mg/dL)	Absolute change at week 12	− 7.42 ± 27.91*	− 5.04 ± 27.38*	− 3.81 ± 23.38
	Absolute change at week 24	− 3.76 ± 31.84	− 7.66 ± 26.80*	− 2.83 ± 26.88
	Absolute change at week 56	− 0.63 ± 36.57	− 1.51 ± 32.05	− 1.97 ± 27.74

Absolute change at week 12 = value at week 12 − value at baseline

Absolute change at week 24 = value at week 24 − value at baseline

Absolute change at week 56 = value at week 56 − value at baseline

*Apo* apolipoprotein A1, *Apo B* apolipoprotein B, *dL* decilitre, *HDL-C* high-density lipoprotein cholesterol, *LDL-C* low-density lipoprotein cholesterol, *mg* milligram, *m* mean, *n* number of patients in each treatment group, *SD* standard deviation, *TC* total cholesterol, *TG* triglyceride, *VLDL-C* very low-density lipoprotein cholesterol

* Significant difference compared to baseline using paired t-test (*p* value < 0.05 which is < 0.016 for each treatment group)

### Safety and tolerability profile

#### Adverse events

During the study (Up to week 56), the incidence of adverse events was similar between all the treatment groups [saroglitazar 2 mg (28.68%), saroglitazar 4 mg (25.91%) and pioglitazone (25.71%)].

Adverse events experienced (In ≥ 2% of patients) in the saroglitazar 2 mg group were [n (%)]: pyrexia 32 (8.42%), headache 22 (5.79%), nasopharyngitis 14 (3.68%), pain 13 (3.42%), cough 9 (2.37%) and hyperchlorhydria 8 (2.11%). In the Saroglitazar 4 mg group the AE’s were headache 18 (4.66%), pyrexia 17 (4.40%) and nausea 8 (2.07%). In the pioglitazone group the AE’s were headache 21 (5.40%), pyrexia 16 (4.11%), diarrhoea 11 (2.83%), asthenia 10 (2.57%) and pain 8 (2.06%). Most of the AE’s were ‘mild’ to ‘moderate’ in severity and were resolved by the completion of the study.

Two serious adverse events (SAEs) were reported in this study. One was a case of acute coronary syndrome and the other coronary artery disease. Detailed causality assessment was performed by the study investigators and both the SAEs were termed ‘Not Related’ to the study drug, by taking into account the pre-existing past medical history of the patients.

#### Body weight

An increase in the body weight was observed in the pioglitazone group at week 12, 24 and 56 (mean change from baseline: 0.01 ± 2.22 kg, 0.17 ± 2.94 kg and 0.41 ± 3.47 kg, respectively). However, a slight decrease in the body weight was observed in saroglitazar 2 mg at week 12, 24 and 56 (mean change from baseline: − 0.07 ± 1.50 kg, − 0.18 ± 2.02 kg and − 0.22 ± 3.20 kg, respectively). Also at week 12 and 56, a decrease in body weight was observed in saroglitazar 4 mg and it was − 0.05 ± 1.67 kg and − 0.01 ± 2.72 kg, respectively.

#### Serum creatinine

No abnormal findings were reported in all the treatment groups for serum creatinine till week 56 (mean: 0.68 mg/dL, 0.75 mg/dL and 0.74 mg/dL for saroglitazar 2 mg; 0.82 mg/dL, 0.82 mg/dL and 0.79 mg/dL for saroglitazar 4 mg and 0.76 mg/dL, 0.73 mg/dL and 0.75 mg/dL for pioglitazone at week 12, 24 and 56, respectively).

#### Hematocrit

No abnormal findings were reported in all the treatment groups for hematocrit till week 56.

### Vital signs

#### Blood pressure

There was a mean reduction in systolic blood pressure in the saroglitazar 2 mg group at week 12, 24 and 56 (mean change from baseline: − 1.13 ± 8.16 mm/Hg, − 1.49 ± 9.08 mm/Hg and − 0.01 ± 8.99 mm/Hg). There was a mean reduction in systolic blood pressure in the saroglitazar 4 mg group at week 12, 24 and 56 (mean change from baseline: − 1.47 ± 8.46 mm/Hg, − 1.41 ± 9.73 mm/Hg and − 0.15 ± 9.53 mm/Hg). A mean reduction in systolic blood pressure was also seen in the pioglitazone group at week 12, 24 and 56 (mean change from baseline: − 1.51 ± 8.39 mm/Hg, − 1.95 ± 9.80 mm/Hg and − 0.02 ± 9.22 mm/Hg).

Similarly, there was a mean reduction in diastolic blood pressure in saroglitazar 2 mg and pioglitazone groups at week 12 and 24 (mean change from baseline: − 0.04 ± 7.34 mm/Hg at week 12 and − 0.40 ± 7.00 mm/Hg at week 24 for saroglitazar 2 mg; − 0.44 ± 6.51 mm/Hg at week 12 and − 0.40 ± 6.91 mm/Hg at week 24 for pioglitazone).

#### Heart rate

There was a mean reduction in heart rate in the saroglitazar 2 mg group at week 12, 24 and 56 (mean change from baseline: − 1.95 ± 6.84 BPM, − 1.88 ± 7.74 BPM and − 1.40 ± 8.68 BPM). There was a mean reduction in heart rate in the saroglitazar 4 mg group at week 12, 24 and 56 (mean change from baseline: − 1.90 ± 7.60 BPM, − 2.36 ± 8.52 BPM and − 1.51 ± 9.64 BPM). A mean reduction in heart rate was also seen in the pioglitazone group at week 12, 24 and 56 (− 1.09 ± 6.95 BPM, − 1.93 ± 7.46 BPM and − 1.33 ± 8.03 BPM).

#### Respiratory rate and body temperature

No abnormal findings were reported for respiratory rate and body temperature from baseline to week 56.

## Discussion

This study was a multi-centric, prospective, randomized, double-blind study to evaluate the efficacy and safety of saroglitazar 2 mg and 4 mg as compared to pioglitazone in type 2 diabetes mellitus patients. In this study, we observed a significant reduction in HbA1c [mean change of − 1.38 ± 1.99, − 1.47 ± 1.92 and − 1.41 ± 1.86, in saroglitazar 2 mg, saroglitazar 4 mg and Pioglitazone groups respectively (*p *< 0.016)] when added to the background stable doses of metformin. Both the strengths of saroglitazar met the primary objective of the study and led to a clinically meaningful reduction of HbA1c levels. This reduction which was evident by week 12 was consistently sustained through week 24 till week 56.

Further, saroglitazar (2 mg and 4 mg) and pioglitazone treatment groups showed statistically significant reduction in FPG and 2 h PPG at week 12, week 24 and week 56 (*p *< 0.016).

Compared with pioglitazone (change from baseline HbA1c), saroglitazar 2 mg was ‘Non Inferior’ at week 24 and week 56. Saroglitazar 4 mg was also ‘Non Inferior’ at week 12, week 24 and week 56 in terms of HbA1c levels. In terms of FPG levels (comparison of change from baseline in FPG), saroglitazar 2 mg was not ‘Non Inferior’ to pioglitazone at week 12 and week 24 but was ‘Non Inferior’ at week 56. Saroglitazar 4 mg was also ‘Non Inferior’ to pioglitazone at week 12, week 24 and week 56 in terms of FPG levels.

In addition, saroglitazar resulted in a statistically significant reduction in lipid parameters (TG, LDL-C, VLDL-C, TC and Non HDL-C) till week 56 treatment duration. On diabetic dyslipidemia, which is typically characterized by increased TG levels and decreased HDL levels, both saroglitazar 2 mg and 4 mg had a favorable effect, wherein there was a clinically meaningful reduction in the TG levels and an increase in the HDL levels. Also both the strengths led to a clinically meaningful reduction in the VLDL-C and LDL-C lipid parameters. In addition, the results of this study are also consistent with the results of the phase 3 clinical trials of saroglitazar in patients with diabetic dyslipidemia for improvement in glycemic and lipid parameters [20, 21]. Saroglitazar is already approved for diabetic dyslipidemia and hypertriglyceridemia with T2DM not controlled by maximum tolerable statin therapy and the results of this study once again validates the results of previous clinical trials [20, 21].

The results obtained in this current phase 3 study are similar to the published literature and previously conducted controlled studies in terms of mean reduction in HbA1c levels (− 1.36%,− 1.4% and − 1.4% mean reductions reported by Einhorn et al., Derosa et al. and Derosa et al.) [26–28].

Kendall et al. evaluated the effect of muraglitazar and pioglitazone in T2DM patients who were inadequately controlled with metformin monotherapy. The results observed that the mean change in HbA1c (%) from baseline of muraglitazar and pioglitazone groups were − 1.14% and − 0.85% at week 24, respectively. At week 12, muraglitazar resulted in a statistically significant reduction in lipid parameters (TG, apolipoprotein B, Non-HDL-C) and an increase in HDL cholesterol level. In addition, a statistically significant reduction in FPG was observed in both the groups [1].

Goldstein et al. investigated the effect of tesaglitazar and pioglitazone in T2DM patients. They observed that the mean change in HbA1c (%) from baseline of tesaglitazar was − 0.3% (5 mg), − 0.59% (1 mg) and − 0.13 (15 mg) and the mean change in HbA1c (%) from baseline of pioglitazone was − 0.42 (30 mg) and − 0.54 (45 mg) at week 24, respectively. Tesaglitazar 1 mg improved TG, HDL-C and non-HDL-C levels compared with all pioglitazone doses at 24 weeks (p < 0.001) [29]. Low-density lipoprotein cholesterol was lower with tesaglitazar for all pioglitazone comparisons (p < 0.05), except for tesaglitazar 0.5 mg versus pioglitazone 15 mg [2].

Stirban et al. evaluated the effect of aleglitazar in T2DM patients who were inadequately controlled with metformin monotherapy. The authors found that the mean change in HbA1c (%) from baseline of aleglitazar and placebo groups were − 0.48% and − 0.01% at week 16, respectively. At week 16, aleglitazar resulted in a statistically significant reduction in lipid parameter (TG) and increased in HDL cholesterol level. In addition, a statistically significant reduction in FPG was observed in aleglitazar group [30].

Compared with other PPAR-α/γ agonists (such as muraglitazar, tesaglitazar and aleglitazar), greater mean HbA1C reductions occurred with saroglitazar. In terms of lipid parameters, saroglitazar resulted in a statistically significant reduction in lipid parameters just like the other glitazars [1, 2, 30]. Additionally, saroglitazar applies its valuable effects on adipose tissue in a rodent model by limiting diet-induced adipose tissue dysfunction, adipocyte hypertrophy, adipocyte cell damage and extracellular matrix deposition in obesity [31] and coadministration of saroglitazar does not cause clinically relevant drug-drug interaction (as per pharmacokinetic data of diverse CYP2C8 substrates) [32].

In May 2006, the clinical development program of muraglitazar and tesaglitazar were discontinued due to safety concerns [14]. Muraglitazar was discontinued due to cardiovascular AEs such as myocardial infarction, stroke, heart failure and tesaglitazar was discontinued due to elevated serum creatinine and a decrease in glomerular filtration rate [14, 17]. In addition, development of aleglitazar was also terminated due to AEs such as heart failure, gastrointestinal bleeding, and renal dysfunction [17, 33]. Saroglitazar is the first and only dual PPAR α/γ agonist (Glitazars) which provides beneficial effects on lipid profile and glycemic control for the treatment of dyslipidemia and diabetes and is in clinical practice [17, 19–21, 34]. Following marketing authorization in India (2013) for diabetic dyslipidemia, saroglitazar has also received approval for marketing as an add-on therapy to metformin for treatment of type 2 diabetes mellitus in India (January 2020) and for Non cirrhotic Non-alcoholic steatohepatitis in March 2020.

PPAR agonists have been reported to inhibit vascular smooth muscle cell proliferation, decrease the risk of thrombosis and suppression of atherosclerosis or restenosis [35–39]. So, PPAR agonists have a potential to improve restoration of the cardiovascular system and its associated cardiovascular risk. Mori et al. observed the improvement of the cardiovascular system and its associated cardiovascular risk in a pre-clinical study. In this pre-clinical study, the effect of PPAR γ agonist (pioglitazone) was studied to evaluate the improvement of cardiac function. The authors investigated the combined administration of pioglitazone and adipose tissue-derived regenerative cells in a rat ischemic cardiomyopathy model. They observed stronger improvement of cardiac function and enhancement of adiponectin paracrine effects [40].

Similarly, Lincoff et al., Woo et al. and Chan et al. observed the improvement of the cardiovascular system and its associated cardiovascular risk in the clinical studies. Lincoff et al. systematically evaluated the effect of pioglitazone on ischemic cardiovascular events. The authors found that pioglitazone is associated with a significantly lower risk of death, myocardial infarction, or stroke among a diverse population of patients with diabetes [41, 42]. Woo et al. evaluated the effect of pioglitazone in acute ischemic stroke patients with diabetes mellitus in a nested case–control study. The authors found that pioglitazone showed significant cardiovascular preventive effect in diabetic patients with acute ischemic stroke [43]. Chan et al. concluded that glitazones as an add-on drug to metformin are associated with reduced major adverse cardiovascular event risk when compared with sulfonylurea added to metformin [44].

Likewise, saroglitazar 2 mg and 4 mg significantly reduced non HDL/HDL ratio and atherogenic index of plasma at week 12 and week 24 compared to baseline in dyslipidemic patients. This indicates that saroglitazar reduces predictor lipid biomarkers of cardiovascular diseases [45]. In addition, several clinical studies investigated the effect of saroglitazar in NAFLD/NASH patients. They concluded that saroglitazar significantly reduced liver fat content, liver stiffness and fibrosis (measured by magnetic resonance imaging-derived proton density-fat fraction and transient elastography) in patients with NAFLD/NASH [46–49]. Overall, as per current literature, saroglitazar has a potential to address the cardiovascular risk associated with high non-HDL-C, high TG, and low HDL-C in patients with diabetic dyslipidemia [50].

In order to assess the potential of saroglitazar for CVD risk reduction, in our previous study (PRESS V), saroglitazar 2 mg and 4 mg showed significant improvement in lipid parameters as compared to baseline. A significant improvement in lipids parameters plays a viable role to safeguard the cardiovascular risk [20]. In the present study, saroglitazar 2 mg and 4 mg resulted in a statistically significant improvement in plasma TG, LDL-C, VLDL-C, TC and Non-HDL cholesterol until week 56. Thus, the results of this study once again validates the results of previous clinical trials in terms of lipid parameters and cardiovascular risk [6, 20].

Khaw et al. demonstrated the association of HbA1c with cardiovascular disease and its risk in diabetes patients. The authors found that an increase in HbA1c of one percentage point was related with cardiovascular risk (coronary heart disease, cardiovascular disease events, and all-cause mortality) [51]. The results of the present study show that the mean change in HbA1c (%) from baseline of Saroglitazar 2 mg and 4 mg treatment groups was − 1.38% and − 1.47% (*p *< 0.016), respectively. Based on current study findings, saroglitazar 2 mg and 4 mg reduced the concentration of HbA1c by > 1 percentage. Hence, saroglitazar has a potential to reduce the cardiovascular risk in T2DM patients.

Most antidiabetic drugs tested in clinical trials reduce HbA1c by 0.5–1.5%, depending on the study design, baseline HbA1c and study population, with combinations resulting in quite similar glycemic outcomes but offering a range of non-glycemic effects and attributes [52]. According to the position statements of the American Diabetes Association and the European Association for the study of Diabetes, the entire clinical picture should be considered so that treatment can be personalized based not only on HbA1c reduction reported in many trials, but also on tolerability, safety, frequency and ease of administration [53]. The results of this current study revealed that a significant reduction in HbA1c in all the treatment groups at week 24 when added to stable doses of metformin. In addition, saroglitazar resulted in a statistically significant reduction in lipid parameters (TG, LDL-C, VLDL-C, TC and Non HDL-C) till week 56. So, saroglitazar can be a viable treatment option for patients of type 2 diabetes mellitus receiving background metformin therapy.

### Safety discussion

The incidence of adverse events (AEs) was similar in all the treatment groups [saroglitazar 2 mg (28.68%), saroglitazar 4 mg (25.91%) and pioglitazone (25.71%)] until week 56. Two subjects reported SAEs during this study, and they were discontinued from the study due to the SAEs. These two SAEs were considered by investigators to be ‘not related’ to study medication.

In this study, no abnormal findings were reported in all the treatment groups for serum creatinine, hematocrit, respiratory rate and body temperature. Increases in mean body weight were seen in patients who received pioglitazone. Weight gain is well known side-effect of PPAR γ agents and may be associated with fluid retention and due to increase in subcutaneous fat. However, slight decrease in body weight was observed in saroglitazar 2 mg (at week 12, 24 and 56) and saroglitazar 4 mg (at week 12 and 56).

Overall, there were no any safety concerns with saroglitazar and it was well tolerated by the patients.

### Limitation

- It is reported that with the usage of pioglitazone, the risk of diabetic macular edema (DME) will increase. However, in this study, the protocol did not factor in this possibility and hence while screening the patients, DME was not ruled out through any diagnostic methodology.

## Conclusions

In conclusion, treatment with saroglitazar 2 mg and 4 mg effectively improved glycemic control and lipid parameters over a 56 week period in patients of type 2 diabetes mellitus receiving background metformin therapy. Based on improvement in glycemic control and lipid parameters, saroglitazar 2 mg and 4 mg have a promising potential to reduce the cardiovascular risk in T2DM patients.

## Data Availability

The datasets used and/or analysed during the current study are available from the corresponding author on reasonable request.

## Abbreviations

ADA: American Diabetes Association
AEs: Adverse events
ALT: Alanine transaminase
ANCOVA: Analysis of co-variance
Apo A1: Apolipoprotein A1
Apo B: Apolipoprotein B
BP: Blood pressure
CI: Confidence intervals
CVDs: Cardiovascular diseases
CDSCO: Central Drugs Standard Control Organization
DCGI: Drugs Controller General of India
DME: Diabetic macular edema
DSMB: Data Safety Monitoring Board
ECG: Electrocardiogram
ECHO: Echocardiography
FPG: Fasting plasma glucose
GCP: Good Clinical Practice
TG: Triglyceride
HbA1c: Glycosylated hemoglobin
HDL-C: High-density lipoprotein cholesterol
ICH: International Council for Harmonisation
ICMR: Indian Council of Medical Research
IRB: Institutional review board
LDL-C: Low-density lipoprotein cholesterol
NAFLD: Nonalcoholic fatty liver disease
NASH: Nonalcoholic steatohepatitis
Non-HDL-C: Non high-density lipoprotein cholesterol
NYHA: New York Heart Association
PPAR: Peroxisome proliferator–activated receptors
PPG: Postprandial plasma glucose
SAE: Serious adverse event
SD: Standard deviation
T2DM: Type 2 diabetes mellitus
UNL: Upper normal limit
VLDL-C: Very low-density lipoprotein cholesterol

## References

[1] Kendall DM, Rubin CJ, Mohideen P, Ledeine JM, Belder R, Gross J, Norwood P, O’Mahony M, Sall K, Sloan G, Roberts A (2006). Improvement of glycemic control, triglycerides, and hdl cholesterol levels with muraglitazar, a dual (α/γ) peroxisome proliferator-activated receptor activator, in patients with type 2 diabetes inadequately controlled with metformin monotherapy: a double-blind, randomized, pioglitazone-comparative study. Diabetes Care.

[2] Bays H, McElhattan J, Bryzinski BS (2007). A double-blind, randomised trial of tesaglitazar versus pioglitazone in patients with type 2 diabetes mellitus. Diabetes Vasc Dis Res.

[3] IDF Diabetes Atlas, Ninth edition; 2019. https://www.diabetesatlas.org/upload/resources/2019/IDF_Atlas_9th_Edition_2019.pdf. Accessed on 07 Mar 2020.

[4] Unoki H, Takahashi A, Kawaguchi T, Hara K, Horikoshi M, Andersen G, Ng DP, Holmkvist J, Borch-Johnsen K, Jørgensen T, Sandbæk A (2008). SNPs in KCNQ1 are associated with susceptibility to type 2 diabetes in East Asian and European populations. Nat Genet.

[5] Tabassum R, Chauhan G, Dwivedi OP, Mahajan A, Jaiswal A, Kaur I, Bandesh K, Singh T, Mathai BJ, Pandey Y, Chidambaram M (2013). Genome-wide association study for type 2 diabetes in Indians identifies a new susceptibility locus at 2q21. Diabetes.

[6] Kaul U, Arambam P, Kachru R, Bhatia V, Diana Y (2019). A prospective, multicentre, single arm clinical study to evaluate the effect of saroglitazar on non high-density lipoprotein cholesterol in patients with diabetic dyslipidemia inadequately controlled with diet, exercise, and statin-the GLIDDER study. J Diabetes Metab..

[7] Yusuf S, Hawken S, Ôunpuu S, Dans T, Avezum A, Lanas F, McQueen M, Budaj A, Pais P, Varigos J, Lisheng L (2004). Effect of potentially modifiable risk factors associated with myocardial infarction in 52 countries (the INTERHEART study): case-control study. Lancet.

[8] Terry T, Raravikar K, Chokrungvaranon N, Reaven PD (2012). Does aggressive glycemic control benefit macrovascular and microvascular disease in type 2 diabetes?: insights from ACCORD, ADVANCE, and VADT. Curr Cardiol Rep.

[9] Zheng Y, Ley SH, Hu FB (2018). Global aetiology and epidemiology of type 2 diabetes mellitus and its complications. Nat Rev Endocrinol.

[10] Saad MF, Greco S, Osei K, Lewin AJ, Edwards C, Nunez M, Reinhardt RR (2004). Ragaglitazar improves glycemic control and lipid profile in type 2 diabetic subjects: a 12-week, double-blind, placebo-controlled dose-ranging study with an open pioglitazone arm. Diabetes Care.

[11] American Diabetes Association (2008). Standards of medical care in diabetes—2008. Diabetes Care.

[12] Saydah SH, Fradkin J, Cowie CC (2004). Poor control of risk factors for vascular disease among adults with previously diagnosed diabetes. JAMA.

[13] Bermudez V, Finol F, Parra N, Parra M, Pérez A, Penaranda L, Vílchez D, Rojas J, Arráiz N, Velasco M (2010). PPAR-γ agonists and their role in type 2 diabetes mellitus management. Am J Ther.

[14] Conlon D (2006). Goodbye glitazars?. Br J Diabetes Vasc Dis.

[15] Henry RR, Lincoff AM, Mudaliar S, Rabbia M, Chognot C, Herz M (2009). Effect of the dual peroxisome proliferator-activated receptor-α/γ agonist aleglitazar on risk of cardiovascular disease in patients with type 2 diabetes (SYNCHRONY): a phase II, randomised, dose-ranging study. Lancet..

[16] Boden G, Laakso M (2004). Lipids and glucose in type 2 diabetes: what is the cause and effect?. Diabetes Care.

[17] Sosale A, Saboo B, Sosale B (2015). Saroglitazar for the treatment of hypertrig-lyceridemia in patients with type 2 diabetes: current evidence. Diabetes Metab Syndr Obes Targets Ther.

[18] Agrawal R (2014). The first approved agent in the Glitazar’s class: saroglitazar. Curr Drug Targets.

[19] Jain MR, Giri SR, Trivedi C, Bhoi B, Rath A, Vanage G, Vyas P, Ranvir R, Patel PR (2015). Saroglitazar, a novel PPARα/γ agonist with predominant PPARα activity, shows lipid-lowering and insulin-sensitizing effects in preclinical models. Pharmacol Res Perspect.

[20] Pai V, Paneerselvam A, Mukhopadhyay S, Bhansali A, Kamath D, Shankar V, Gambhire D, Jani RH, Joshi S, Patel P (2014). A multicenter, prospective, randomized, double-blind study to evaluate the safety and efficacy of saroglitazar 2 and 4 mg compared to pioglitazone 45 mg in diabetic dyslipidemia (PRESS V). J Diabetes Sci Technol.

[21] Jani RH, Pai V, Jha P, Jariwala G, Mukhopadhyay S, Bhansali A, Joshi S (2014). A multicenter, prospective, randomized, double-blind study to evaluate the safety and efficacy of Saroglitazar 2 and 4 mg compared with placebo in type 2 diabetes mellitus patients having hypertriglyceridemia not controlled with atorvastatin therapy (PRESS VI). Diabetes Technol Ther.

[22] LaCivita KA, Villarreal G (2002). Differences in lipid profiles of patients given rosiglitazone followed by pioglitazone. Curr Med Res Opin.

[23] Van Wijk JP, De Koning EJ, Martens EP, Rabelink TJ (2003). Thiazolidinediones and blood lipids in type 2 diabetes. Arterioscler Thromb Vasc Biol.

[24] Boyle PJ, King AB, Olansky L, Marchetti A, Lau H, Magar R, Martin J (2002). Effects of pioglitazone and rosiglitazone on blood lipid levels and glycemic control in patients with type 2 diabetes mellitus: a retrospective review of randomly selected medical records. Clin Ther.

[25] Goldberg RB, Kendall DM, Deeg MA, Buse JB, Zagar AJ, Pinaire JA, Tan MH, Khan MA, Perez AT, Jacober SJ (2005). A comparison of lipid and glycemic effects of pioglitazone and rosiglitazone in patients with type 2 diabetes and dyslipidemia. Diabetes Care.

[26] Einhorn D, Rendell M, Rosenzweig J, Egan JW, Mathisen AL, Schneider RL (2000). Pioglitazone hydrochloride in combination with metformin in the treatment of type 2 diabetes mellitus: a randomized, placebo-controlled study. Clin Ther.

[27] Derosa G, Maffioli P, Salvadeo SA, Ferrari I, Ragonesi PD, Querci F, Franzetti IG, Gadaleta G, Ciccarelli L, Piccinni MN, D’Angelo A (2010). Effects of sitagliptin or metformin added to pioglitazone monotherapy in poorly controlled type 2 diabetes mellitus patients. Metabolism..

[28] Derosa G, D’Angelo A, Ragonesi PD, Ciccarelli L, Piccinni MN, Pricolo F, Salvadeo SA, Montagna L, Gravina A, Ferrari I, Paniga S (2007). Metabolic effects of pioglitazone and rosiglitazone in patients with diabetes and metabolic syndrome treated with metformin. Intern Med J.

[29] Goldstein BJ, Rosenstock J, Anzalone D, Tou C, Peter Öhman K (2006). Effect of tesaglitazar, a dual PPARα/γ agonist, on glucose and lipid abnormalities in patients with type 2 diabetes: a 12-week dose-ranging trial. Curr Med Res Opin.

[30] Stirban AO, Andjelkovic M, Heise T, Nosek L, Fischer A, Gastaldelli A, Herz M (2016). Aleglitazar, a dual peroxisome proliferator-activated receptor-α/γ agonist, improves insulin sensitivity, glucose control and lipid levels in people with type 2 diabetes: findings from a randomized, double-blind trial. Diabetes Obes Metab.

[31] Kumar D, Goand UK, Gupta S, Shankar K, Varshney S, Rajan S, Srivastava A, Gupta A, Vishwakarma AL, Srivastava AK, Gaikwad AN (2018). Saroglitazar reduces obesity and associated inflammatory consequences in murine adipose tissue. Eur J Pharmacol.

[32] Giri P, Delvadia P, Ladani MK, Prajapati N, Gupta L, Patel N, Joshi V, Giri S, Jain MR, Srinivas NR, Patel PR (2019). Lack of inhibition of CYP2C8 by saroglitazar magnesium: in vivo assessment using montelukast, rosiglitazone, pioglitazone, repaglinide and paclitaxel as victim drugs in Wistar rats. Eur J Pharm Sci.

[33] Lincoff AM, Tardif JC, Schwartz GG, Nicholls SJ, Rydén L, Neal B, Malmberg K, Wedel H, Buse JB, Henry RR, Weichert A (2014). Effect of aleglitazar on cardiovascular outcomes after acute coronary syndrome in patients with type 2 diabetes mellitus: the AleCardio randomized clinical trial. JAMA.

[34] Joshi SR (2015). Saroglitazar for the treatment of dyslipidemia in diabetic patients. Expert Opin Pharmacother.

[35] Gizard F, Amant C, Barbier O, Bellosta S, Robillard R, Percevault F, Sevestre H, Krimpenfort P, Corsini A, Rochette J, Glineur C (2005). PPARα inhibits vascular smooth muscle cell proliferation underlying intimal hyperplasia by inducing the tumor suppressor p16 INK4a. J Clin Investig.

[36] Ringseis R, Müller A, Herter C, Gahler S, Steinhart H, Eder K (2006). CLA isomers inhibit TNFα-induced eicosanoid release from human vascular smooth muscle cells via a PPARγ ligand-like action. Biochimica et Biophysica Acta BBA General Subjects..

[37] Xiong C, Mou Y, Zhang J, Fu M, Chen YE, Akinbami MA, Cui T (2005). Impaired expression of PPARγ protein contributes to the exaggerated growth of vascular smooth muscle cells in spontaneously hypertensive rats. Life Sci.

[38] Gervois P, Vu-Dac N, Kleemann R, Kockx M, Dubois G, Laine B, Kosykh V, Fruchart JC, Kooistra T, Staels B (2001). Negative regulation of human fibrinogen gene expression by peroxisome proliferator-activated receptor α agonists via inhibition of CCAAT box/enhancer-binding protein β. J Biol Chem.

[39] Koh KK, Ahn JY, Han SH, Jin DK, Kim HS, Lee KC, Shin EK, Sakuma I (2004). Effects of fenofibrate on lipoproteins, vasomotor function, and serological markers of inflammation, plaque stabilization, and hemostasis. Atherosclerosis..

[40] Mori D, Miyagawa S, Matsuura R, Sougawa N, Fukushima S, Ueno T, Toda K, Kuratani T, Tomita K, Maeda N, Shimomura I (2019). Pioglitazone strengthen therapeutic effect of adipose-derived regenerative cells against ischemic cardiomyopathy through enhanced expression of adiponectin and modulation of macrophage phenotype. Cardiovasc Diabetol.

[41] Lincoff AM, Wolski K, Nicholls SJ, Nissen SE (2007). Pioglitazone and risk of cardiovascular events in patients with type 2 diabetes mellitus: a meta-analysis of randomized trials. JAMA.

[42] Dormandy JA, Charbonnel B, Eckland DJ, Erdmann E, Massi-Benedetti M, Moules IK, Skene AM, Tan MH, Lefèbvre PJ, Murray GD, Standl E (2005). Secondary prevention of macrovascular events in patients with type 2 diabetes in the PROactive Study (PROspective pioglitAzone Clinical Trial In macroVascular Events): a randomised controlled trial. Lancet..

[43] Woo MH, Lee HS, Kim J (2019). Effect of pioglitazone in acute ischemic stroke patients with diabetes mellitus: a nested case–control study. Cardiovasc Diabetol.

[44] Chan CW, Yu CL, Lin JC, Hsieh YC, Lin CC, Hung CY, Li CH, Liao YC, Lo CP, Huang JL, Lin CH (2018). Glitazones and alpha-glucosidase inhibitors as the second-line oral anti-diabetic agents added to metformin reduce cardiovascular risk in type 2 diabetes patients: a nationwide cohort observational study. Cardiovasc Diabetol.

[45] Krishnappa M, Shah M, Parmar K, Joshi S, Maroo S, Parmar D (2019). Saroglitazar reduces predictor lipid biomarkers of cardiovascular diseases. Atherosclerosis..

[46] Dadhich SK. Efficacy and safety of saroglitazar in nonalcoholic fatty liver disease patients at 1 year: an investigator initiated study: 1034. Am J Gastroenterol. 2019;114 (2019 ACG Annual Meeting Abstracts): S591. https://journals.lww.com/ajg/Fulltext/2019/10001/Efficacy_and_Safety_of_Saroglitazar_in.1034.aspx. Accessed on 19 May 2020.

[47] Chaudhuri S. Efficacy and safety of saroglitazar in management of NAFLD patients using transient elastography: a single center observational study. https://www.postersessiononline.eu/173580348_eu/congresos/NAFLD2019/aula/-P01_19_NAFLD2019.pdf. Accessed on 19 May 2020.

[48] Goyal O, Goyal P, Nauhria S, Kaur J, Kumar P, Chhina RS (2019). Saroglitazar improves transaminases and transient elastography in patients with diabetic dyslipidemia and non-alcoholic fatty liver disease. J Gastroenterol Hepatol..

[49] Samer Gawrieh, Mazen Noureddin, Nicole M. Loo, Rizwana Mohseni, Vivek R. Awasty, Kenneth Cusi, Kris V. Kowdley, Michelle Lai, Eugene R. Schiff, Deven V. Parmar, Pankaj R. Patel and Naga P. Chalasani. Phase 2, prospective, multicenter, double-blind, randomized study of saroglitazar magnesium 1 mg, 2 mg or 4 mg versus placebo in patients with nonalcoholic fatty liver disease and/or nonalcoholic steatohepatitis (EVIDENCES IV). The American Association for the Study of Liver Diseases. Late-breaking Abstracts; 2019. https://www.aasld.org/sites/default/files/2019-10/2019-TLM-LateBreakingAbstracts.pdf#page=17&zoom=100,45,168. Accessed 25 Apr 2020.

[50] Kaul U, Parmar D, Manjunath K, Shah M, Parmar K, Patil KP, Jaiswal A (2019). New dual peroxisome proliferator activated receptor agonist—Saroglitazar in diabetic dyslipidemia and non-alcoholic fatty liver disease: integrated analysis of the real world evidence. Cardiovasc Diabetol.

[51] Khaw KT, Wareham N, Bingham S, Luben R, Welch A, Day N (2004). Association of hemoglobin A1c with cardiovascular disease and mortality in adults: the European prospective investigation into cancer in Norfolk. Ann Intern Med.

[52] Little RR, Rohlfing CL (2013). The long and winding road to optimal HbA1c measurement. Clin Chim Acta.

[53] Riddle MC (2018). American diabetes association standards of medical care in diabetes. Diabetes Care.

